# Safety of urethral preservation using urethral frozen section analysis in radical cystectomy

**DOI:** 10.1002/bco2.377

**Published:** 2024-05-30

**Authors:** Yuto Hattori, Akihiko Nagoshi, Tasuku Fujiwara, Takanari Kambe, Yuta Mine, Hiroki Hagimoto, Yohei Abe, Daisuke Yamashita, Naofumi Tsutsumi, Noboru Shibasaki, Toshinari Yamasaki, Mutsushi Kawakita

**Affiliations:** ^1^ Department of Urology Kobe City Medical Centre General Hospital Kobe Japan; ^2^ Department of Pathology Kobe City Medical Centre General Hospital Kobe Japan

**Keywords:** cystectomy, frozen sections, recurrence, urethra, urinary bladder neoplasms

## Abstract

**Background:**

The objective of this study is to assess whether urethral preservation can be performed safely using frozen section analysis (FSA) of the urethral stump on urethral recurrence after radical cystectomy.

**Methods:**

Between June 2012 and July 2022, we investigated consecutive male patients who underwent urethral FSA during radical cystectomy for urothelial carcinoma. For FSA‐abnormal cases, urethrectomy was performed, and for FSA‐normal cases, the urethra was preserved. The diagnostic accuracy of FSA was assessed in comparison with the pathological findings of the permanent sections of the same tissue. Postoperatively, computed tomography and urinary cytology were performed as routine surveillance of recurrence.

**Results:**

Of the 77 patients included in this study, three patients with abnormal FSA underwent concurrent urethrectomy. The negative predictive value of urethral FSA was 100%. With a median postoperative follow‐up of 38 months (interquartile ranges 21–71), no urethral recurrence was observed.

**Conclusions:**

FSA may be useful in determining the indication for urethrectomy.

## 1 INTRODUCTION

Radical cystectomy (RC) with lymphadenectomy following neoadjuvant chemotherapy is the standard of care for muscle‐invasive bladder cancer. Due to the multifocal and metachronous nature of urothelial carcinoma, recurrence of remnant urothelium occasionally occurs after RC. Despite the low rate of urethral recurrence (1–6%), it is clinically important as it affects cancer‐specific survival.^1^

Historically, prophylactic urethrectomy has been performed during RC to prevent urethral recurrence. Although current guidelines do not recommend routine prophylactic urethrectomy, there are no consensus criteria for its indications.^2^ Similarly, although the usefulness of frozen section analysis (FSA) of the urethral stump during RC has been reported, its role is still ambiguous.^3^, ^4^, ^5^

At our hospital, the indication for urethrectomy is determined according to the results of the urethral FSA during RC. In this study, we retrospectively evaluated our urethral management strategies based on FSA for preventing urethral recurrence.

## 2 PATIENTS AND METHODS

Between June 2012 and July 2022, 77 male patients who underwent RC with FSA at our hospital were included in this retrospective study. All patients underwent transurethral resection prior to RC and were histologically confirmed to have urothelial carcinoma. Transurethral resection biopsy of the prostatic urethra was performed in cases with suspected carcinoma in situ (CIS), tumour of the bladder neck or abnormal findings of the prostatic urethra. To obtain a urethral sectional specimen for FSA, the bulbar urethra was retracted as far as possible (3–4 cm) into the pelvis, and then, Hem‐o‐lok® were applied to the bulbar and membranous urethra and cut between them. If the FSA was normal, the urethra was preserved; otherwise, a concurrent urethrectomy via perineal approach was performed. Thereafter, the pelvic floor was closed with a 2‐0 barbed suture.

According to our protocol, the patients were routinely followed up every 3 months for the first 2 years after surgery, then every 6 months for the next 3 years and annually thereafter. Physical examination, blood and urine tests, urinary cytology and computed tomography were performed during these follow‐up visits. In the cases of nonorthotopic urinary diversion, urethral wash cytology was conducted annually.

Descriptive statistics were reported as medians and interquartile ranges for continuous variables and frequencies and proportion for categorical variables. Sensitivity and specificity, positive predictive value (PPV) and negative predictive value (NPV) of FSA were calculated by comparing them with the corresponding permanent sections as reference. The Kaplan–Meier method was used to calculate recurrence‐free survival and overall survival. Statistical analyses were performed using EZR, version 1.53.

## 3 RESULTS

The patient demographic and clinical characteristics are summarized in Table 1. The median age was 73 years. There were 15 bladder neck tumours and 18 multifocal tumours. Of the 37 patients who underwent prostatic urethral biopsy, 13 had prostatic urethral involvement; stromal invasion and CIS were detected in nine and four cases, respectively. There were 44 cases with at least one feature of bladder neck lesion, multifocal tumours or prostatic urethral involvement.

**TABLE 1:** Patient demographic and clinical characteristics.

Variables	*n* = 77	
Median age, years (IQR)	73	(68–78)
Median ASA‐PS (IQR)	2	(2–3)
Clinical T‐stage (%)		
≤cT1	12	(15.6)
cT2	48	(62.3)
cT3	13	(16.9)
cT4	4	(5.2)
Clinical N‐stage (%)		
cN0	66	(85.7)
cN1	5	(6.5)
cN2	6	(7.8)
Primary tumour location (%)		
Anterior wall	2	(2.6)
Dome	6	(7.8)
Lateral wall	26	(33.8)
Trigone	20	(26.0)
Posterior wall	8	(10.4)
Bladder neck	15	(19.5)
Prostatic urethral invasion (%)^a^	13	(16.9)
Neoadjuvant chemotherapy (%)	56	(72.7)
Type of operation (%)		
Open RC	5	(6.5)
Laparoscopic RC	23	(29.9)
Robot‐assisted RC	49	(63.6)
Type of urinary diversion (%)		
Ileal conduit	54	(70.1)
Cutaneous diversion	7	(9.1)
Neobladder	10	(13.0)
None	6	(7.8)
Pathological T‐stage (%)		
pT0	24	(31.2)
pTa/Tis/T1	15	(19.5)
pT2	14	(18.2)
pT3	19	(24.7)
pT4	5	(6.5)
Pathological N‐stage (%)		
pNx	4	(5.2)
pN0	52	(67.5)
pN1	9	(11.7)
pN2	6	(7.8)
pN3	6	(7.8)
Multifocal diseases (%)	18	(23.4)
Carcinoma in situ (%)	23	(29.9)
Median follow‐up, months (IQR)	38	(21–71)
Urethral recurrence (%)	0	(0)
Non‐urothelial recurrence (%)	17	(22.1)
Deaths (%)		
All causes	21	(27.3)
Due to urothelial carcinoma	12	(15.6)

Abbreviations: ASA‐PS, American Society of Anesthesiologists physical status; IQR, interquartile range; RC, radical cystectomy.

^a^ Thirty‐seven patients underwent prostatic urethral biopsy.

Seventy‐four patients with normal FSA preserved the urethra. Three FSA‐abnormal patients underwent concurrent urethrectomy. FSAs in all cases were consistent with permanent specimens; thus, the sensitivity, specificity, PPV and NPV were all 100% (Tables 2 and 3). With a median follow‐up of 38 (interquartile range 21–71) months, recurrences of urothelial carcinoma were found in 17 patients, while no case had a urethral recurrence. There were 21 deaths, including 12 cancer‐specific deaths. The 5 year recurrence‐free survival rate and overall survival rate were 76.1% and 63.8%, respectively.

**TABLE 2:** Clinical characteristics of three patients with abnormal frozen section analysis of urethral stump.

	Tumour location	Prostatic urethral invasion	Multifocality	Concomitant CIS	Pathology of frozen section	Pathology of permanent section	Pathology of anterior urethra	Follow‐up
1	Dome	NA	(−)	(+)	UC	UC	NEM	3 months; DOD
2	Trigone	NA	(−)	(+)	Atypical cell	Atypical cell	Denudation	23 months; DOD
3	Lateral wall	(−)	(−)	(−)	Suspicious for UC	Suspicious for UC	NEM	28 months; AWD

Abbreviation: CIS, carcinoma in situ.

**TABLE 3:** Correlation of the frozen section and the corresponding permanent sections of the urethral stump.

			Permanent section analysis		
			Abnormal		Normal
			Urothelial carcinoma	Atypical	Normal
Frozen section analysis	Abnormal	Urothelial carcinoma	2	0	0
		Atypical	0	1	0
	Normal	Normal	0	0	74

## 4 DISCUSSION

Concurrent urethrectomy with RC is one of the options of urethral management for preventing urethral recurrence. However, in recent years, with the increase in orthotopic urinary diversions, the benefits of prophylactic urethrectomy have become questionable and are only performed in selected cases.^6^ Nevertheless, there are no established indications for prophylactic urethrectomy. The role of urethral FSA also remains ambiguous. In our study, the accuracy of FSA was sufficiently high as 100% of NPV with no urethral recurrence during the 38 month observation period, indicating that urethrectomy based on the FSA is reasonable for urethral management during RC.

Urethral recurrence has been reported to have many risk factors, such as multiple lesions, tumour location (bladder neck or prostatic urethra), pathological T‐stage, nonorthotopic diversions and concomitant CIS,^1^, ^7^ among which prostate involvement is the most significant risk factor.^8^ However, von Rundstedt et al. reported that the transurethral biopsy of the prostatic urethra had a low PPV.^9^ Furthermore, Lebret et al. showed that favourable results were obtained when FSA was normal, even in the case of prostatic urethral involvement.^4^ Therefore, cases with prostatic urethral lesions should not be excluded from orthotopic urinary diversion and FSA confirmation is recommended.^8^, ^9^

In Japan, it is relatively common to perform prophylactic urethrectomy for nonorthotopic urinary diversion, as suggested by the Japanese Urological Association guideline.^10^ In contrast, the European Association of Urology guidelines do not recommend removal of the entire urethra in all cases of RC.^2^ There is no consensus on which cases should undergo urethrectomy. Recently, Laukhtina et al. reported that prophylactic urethrectomy is beneficial in the presence of risk factors such as bladder neck invasion, multifocality and prostatic urethra involvement.^7^ However, in our study, among 44 cases that had one or more of the above risk factors, only two cases had abnormal FSA. Performing prophylactic urethrectomy in all high‐risk cases, therefore, sounds excessive.

Diagnostic limitations of FSA have been noted, including nuclear enlargement, staining irregularity and tissue destruction.^11^ In a recent systematic review, the sensitivity, specificity, PPV and NPV of FSA were 83% (95% CI 0.38–0.98), 95% (95% CI 0.91–0.97), 62% (95% CI 0.53–0.71) and 99% (95% CI 0.92–0.99), respectively.^12^ Considering the low PPV, performing urethrotomy in FSA‐positive cases may be overtreatment. On the other hand, because of the very high NPV consistent with our results, the urethra can be safely preserved in FSA‐negative cases. Regarding cases where the FSA was atypical, Gordetsky et al. reported that 16.7% were diagnosed with CIS on a permanent specimen.^11^ They suggest obtaining an additional specimen in such cases, but unlike the ureters, this is technically difficult in the urethra. Given these considerations, our strategy of urethral resection unless the FSA is normal may be reasonable.

Guideline recommendations also vary by the type of urinary diversion. In the neobladder, confirmation of a normal FSA of the urethral stump is recommended. In the nonorthotopic urinary diversion, on the other hand, there is no recommendation for FSA about preserving the urethra. Although patients with nonorthotopic urinary diversion are at higher risk of urethral recurrence,^13^, ^14^ one protocol has been proposed in which FSA may be omitted.^3^ One of the features of nonorthotopic urinary diversion is that early detection of urethral recurrence is difficult as urine cannot pass through the urethra. Clark et al. reported that only about one third of cases of urethral recurrence could be diagnosed by urethral wash cytology and that most diagnoses were symptom‐based.^15^ Unfortunately, patients with symptomatic urethral recurrence have a poorer prognosis than asymptomatic patients (median OS 4.8 vs. 8.3 years: *p* = 0.05).^16^ Therefore, even in nonorthotopic urinary diversions, a cautious evaluation of the indications for urethral preservation is necessary, and the FSA may be helpful in making this determination.

This study has a few limitations. First, it is a single‐arm, single‐centre retrospective study, with a small size and a short follow‐up period. Especially because there are only three cases of abnormal FSA, the PPV is not a reliable value. Second, in addition to the insufficient diagnostic accuracy of urethral wash cytology, some cases of urethral wash cytology were not performed according to our protocol. This may have resulted in cases of asymptomatic urethral recurrence being overlooked. Third, neoadjuvant chemotherapy has been reported to reduce urethral recurrence, and the lower rate of urethral recurrence compared with previous reports may be related to the high rate of neoadjuvant chemotherapy in our study.^17^ Despite these limitations, the FSA of the urethral stump is an easy‐to‐perform and high NPV that has the potential to play an important role in urethral management during RC, regardless of the type of urinary diversion.

In conclusion, the FSA of the urethral stump has a high NPV and may be helpful in selecting candidates for prophylactic urethrectomy in RC.

## AUTHOR CONTRIBUTIONS

All authors contributed to the study conception and design. The first draft of the manuscript was written by Yuto Hattori. Previous versions of the manuscript were reviewed and edited by all authors. The study was supervised by Mutsushi Kawakita and Toshinari Yamasaki throughout the process. All authors read and approved the final manuscript.

## CONFLICT OF INTEREST STATEMENT

The authors have no conflicts of interest to disclose.
